# Ring Counter Based ATPG for Low Transition Test Pattern Generation

**DOI:** 10.1155/2015/729165

**Published:** 2015-05-14

**Authors:** V. M. Thoulath Begam, S. Baulkani

**Affiliations:** ^1^Department of ICE, Anna University, Chennai 600 025, India; ^2^Department of ECE, Government College of Engineering, Tirunelveli 627 002, India

## Abstract

In test mode test patterns are applied in random fashion to the circuit under circuit. This increases switching transition between the consecutive test patterns and thereby increases dynamic power dissipation. The proposed ring counter based ATPG reduces vertical switching transitions by inserting test vectors only between the less correlative test patterns. This paper presents the RC-ATPG with an external circuit. The external circuit consists of XOR gates, full adders, and multiplexers. First the total number of transitions between the consecutive test patterns is determined. If it is more, then the external circuit generates and inserts test vectors in between the two test patterns. Test vector insertion increases the correlation between the test patterns and reduces dynamic power dissipation. The results prove that the test patterns generated by the proposed ATPG have fewer transitions than the conventional ATPG. Experimental results based on ISCAS'85 and ISCAS'89 benchmark circuits show 38.5% reduction in the average power and 50% reduction in the peak power attained during testing with a small size decoding logic.

## 1. Introduction

Built-in self-test (BIST) is a design-for-test (DFT) technique in which testing is achieved through built-in hardware features. The steps in a typical BIST approach are as follows:

1. on-chip test pattern generation (TPG);
2. application of patterns to the circuit under test (CUT);
3. analysis of CUT responses via on-chip output response analyzer (ORA);
4. making decision whether chip is faulty or not.

Efficient TPG design is related to step (1) and it is an important subject in BIST.

Generation of test vector sequences with low power consumption and high fault coverage in minimal hardware size is the main objective of this proposed approach.

In recent years, power consumption during testing has become an important issue in test manufacturing because high circuit activity rate during test generation and/or high fan-out of BIST components may result in passing the package power consumption limits which in turn may risk the health of the test [1, 2].

Power consumption of VLSI during test application can be as high as 200% of that in normal mode as reported in [2, 3].

Therefore, reducing power consumption in test mode is becoming an important objective in circuit design. The techniques used in [4, 5] usually incur fewer mathematical constraints but require a variable-length encoding of the test cubes, and this complicates the communication between the ATE and the chips.

Thermal-aware methodology [6] can save 17.5% more power consumed by the repeaters but it allows delay. In [7, 8], low transition test vector is inserted between two consecutive patterns, even if there are a few number of transitions. This method increases testing time. The hybrid method [9] only concentrated on avoiding repeated pattern generation and the methods in [10, 11] are used for reduction of the memory size needed for test pattern storage.

In the proposed scheme, the multiple seeds are stored in ROM, which are used to skip the nondetecting vectors. For each seed, the inputs of CUT are divided into two groups (partial-acting inputs and partial-freezing inputs) according to determined number of transitions. If number of transitions increased, test vectors are generated using random bits insertion and frozen partial test pattern. This decreases vertical transitions, reducing dynamic power dissipation.

In [12–14] a twisted ring counter along with some reseeding logic is employed to generate the required patterns. A control unit is used to load seed patterns into the input scan registers and to perform the TRC operations so as to generate more patterns from the seeds. But the experimental results show that long test application time is still needed to achieve complete fault coverage.

The remainder of this paper is organized as follows. In Section 2, prior work regarding TPG is given.  Section 3 describes the proposed ATPG circuit and the state diagram of FSM which is used to implement the proposed scheme. It is followed by hardware implementation small size (8-bit CUT) circuit in Section 4.  Section 5 presents experimental results for the ISCAS benchmark circuits.  Section 6 concludes the paper.

## 2. Prior Work

Many schemes were proposed for generating low power test patterns using LFSR as ATPG. In some methods two clocks are used which also increase power dissipation. The method using RSIC (random single input change) test generation generates low transition test patterns but at additional cost for reducing power.

In one method using LFSR as TPG, flip flops are divided into two sets which make the circuit complex. In this paper, ring counter is used as test pattern generator and an external circuit is added with TPG for reducing switching transitions makes the circuit simple.

## 3. Proposed RC-ATPG

**Figure 1:**
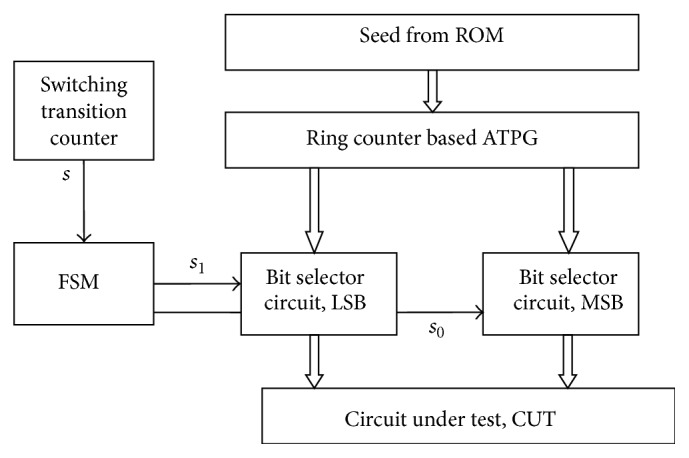
General architecture of the proposed method.

The general architecture of the proposed scheme is shown in Figure 1 with ROM and CUT. The seeds are fed from ROM to ATPG whenever it is needed. The TPG is nothing but a ring counter (RC) which generates test patterns. Each test pattern is applied to switching transition counter (STC) circuit to determine its number of switching transitions before it is sent to CUT. Depending upon the number of transitions FSM generates outputs. The outputs are applied as select lines to bit selector circuits. If the test pattern has more number of transitions, test vectors are inserted by BSC which reduces vertical transitions.

With regular interval, seed value is applied from ROM, to avoid the same pattern generation. Seed value is applied in parallel which reduces the time for seed application. Seed value is obtained using any optimization algorithm. Normally in test pattern generation genetic algorithm is used as optimization algorithm.

In this RC-based TPG the D-FFs are replaced by modified D-FFs (Figure 3). Initially each test pattern is equally divided into two parts as least significant bytes and most significant bytes. Half of the flip flop outputs, that is, LSB of the test pattern, are applied to CUT through bit selector LSB circuit. BSC generates partial insertion bits. The remaining (MSB) half of the test pattern must be frozen during test vector insertion using bit selector MSB circuit.

During test vector insertion, LSB is replaced by random bits with frozen MSB. First test vector is the combination of present MSB with random bits as LSB. In the next clock pulse actual LSB of new or next test pattern is applied with the same MSB. In both clock pulses (random bit insertion and original LSB of TP) MSB of test pattern is frozen. In the third pulse next MSB is applied.

In this proposed scheme, all inputs are divided into two groups. One part is the partial-freezing bits, and the other part is the partial insertion bits. In order to implement the proposed scheme, external circuit (STC, FSM, and BSC) is included with RC-based TPG.

### 3.1. RC-ATPG

Normally, ring counter is constructed by any type of flip flops in which the previous output of flip flop is applied as input to the successive flip flop and the last flip flop output is connected with the first flip flop input.

**Figure 2:**
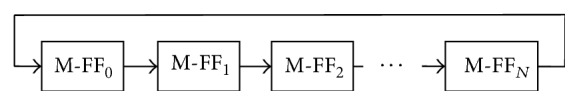
Ring counter ATPG.

**Figure 3:**
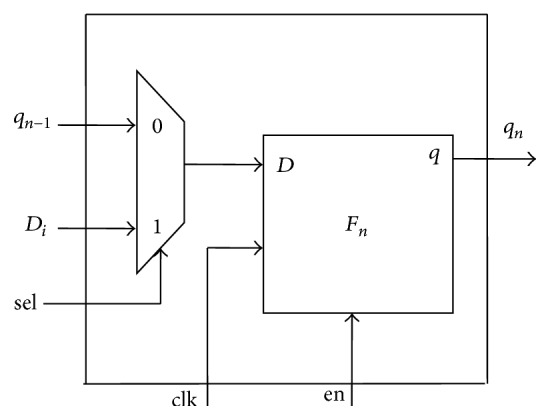
Modified flip flop.

The proposed TPG is implemented using ring counter, which is constructed by using modified D-flip flops (see Figure 2). Each D-FF is combined with multiplexer which is known as modified flip flop. The initial value (seed) is applied to TPG to start test pattern generation. The operation of the register is deterministic; the stream of values produced by the register is completely determined by its current (or previous) state. The output of TPG is directly connected with first FF, which forms a ring counter (RC).

Modified flip flop (M-FF) is the combination of D-FF and multiplexer. It has 4 inputs and only one output. Select line of MUX is used to input the seed value or for ring operation which generates test patterns.

M-FF has five input lines and one output line. It works as in Table 1. Multiplexer selects either seed input or next test pattern bit. The output of multiplexer is applied to D-FF. Flip flop with enable signal is used here. During test vector insertion, the test pattern generation must be hold using the enable signal.

Seed is stored in ROM and applied in parallel by asserting the select input of M-FF as “1”. If the select input of M-FF is “0” then the ring counter starts to generate test patterns using ring operation. With regular interval seed must be applied from ROM to avoid the same test pattern generation. Seeds are also used to generate test patterns that have high fault coverage.

Here each flip flop is replaced by modified flip flop. Initially the seed value is applied by asserting the select line which is equal to logic “1”. Then the select line is deserted and the ring operation is started which generates test patterns. The test patterns are applied through BSC/FC circuit (which reduces vertical switching transitions) to CUT.

### 3.2. Switching Transition Counter

Switching transition counter consists of XOR gates, full adders, and an OR gate. Each consecutive test pattern bit is applied to XOR gate. If the test bits are not the same then the XOR gate output is 1. These 1's are added using full adders.

More numbers of 1's give carry output of full adder equal to 1. More numbers of carry = 1 indicate that the number of transitions between consecutive test patterns is more. The carry output of full adders is given to an OR gate. The OR gate output is applied as input to the FSM.

XOR gates are used to check the transition between horizontal bits. The number of required XOR gates is equal to *N* − 1 where “*N*” is number of bits in the test pattern. The carry outputs of all full adders are ORed to check the total number transitions.

**Figure 4:**
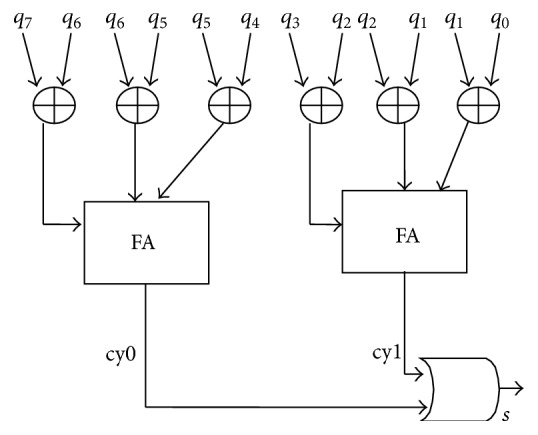
Switching transition counter.

Figure 4 shows ST counter for an 8-bit TPG circuit. The number of XOR gates, full adders, and OR gate depends on the number of CUT inputs and it can be reduced according to our requirement. If the number of test pattern bits is more, then the successive carry outputs are AND ed before applied to the OR gate.

### 3.3. Finite State Machine

**Figure 5:**
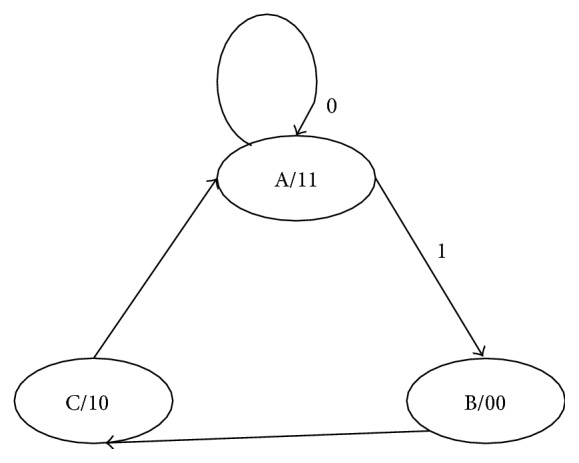
State diagram of FSM.

FSM has a single input, three outputs (*s*_0_, *s*_1_, and en), and 3 states. State diagram of FSM is shown in Figure 5. Output of STC is the input for FSM. It generates outputs *s*_0_ and *s*_1_ which are the select line for BSC and freezer multiplexer. The three states are A, B, C and the initial state of FSM is A.

Depending on the input “*s*,” FSM changes its states. The outputs of FSM are applied as select inputs to BSC. During state A, the outputs are *s*_1_ *s*_0_ = 11. For B state *s*_1_ *s*_0_ = 00 and for C, *s*_1_ *s*_0_ = 10. In state A new test pattern is generated and its transitions are calculated.

More numbers of transitions are indicated by the input *s* equal to 1 from STC circuit.  Table 2 shows the function performed at each state of FSM. During B state random bits are inserted in LSB portion of test pattern. In B and C states partial pattern (MSB) is frozen. The above process is done for each pattern that is generated by ring counter. Test vectors are inserted between the consecutive patterns which are having high transitions.

The function performed in each state of FSM is as follows:

 
*State A: s*_1_ *s*_0_ = 11. The generated new test pattern is sent to CUT directly, if it has less vertical switching transitions.
 
*State B: s*_1_ *s*_0_ = 00. LSB of test pattern is filled with random bits and MSB of present test pattern is frozen.
 
*State C: s*_1_ *s*_0_ = 10. MSB of present test pattern is frozen and LSB is replaced by the new test pattern bits.
 
*State A: s*_1_ *s*_0_ = 11. The MSB is replaced by new test pattern and LSB is kept as it is.

FSM generates enable signal also. This enable output is given to each M-FF, to disable test pattern generation during test vector insertion. Enable is logic “0” in states B and C. At state A, it is logic “1” which starts the test pattern generation.

### 3.4. Bit Selector Circuit

**Figure 6:**
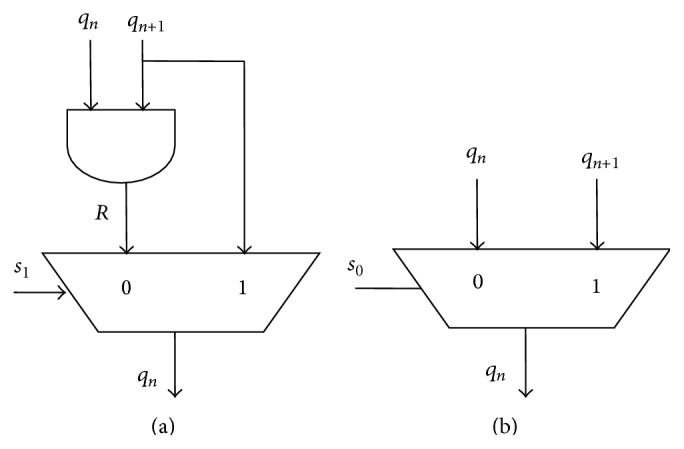
(a) Bit selector circuit, LSB. (b) Bit selector circuit, MSB.

BSC is used for test vector generation and insertion. Separate circuit arrangement is used for LS bits and MS bits of TPG outputs (see Figures 6(a) and 6(b)). For 8-bit TPG the least 4 FF outputs are connected with BSC-LSB and remaining most significant output bits are applied to BSC-MSB circuits.

BSC-LSB is constructed by using 2 : 1 multiplexers with simple AND gate. The output line *s*_1_ from FSM is applied as select input. LSB of each test vector is either next TP bit or random bit. Random bit “*R*” is the AND gate output of present and next test pattern bits (*q*_*n*_, *q*_*n*+1_). For only one clock pulse the LSB is replaced with random bits. In next two pulses LSB is filled with new test pattern bits.

In BSC-MSB only one 2 : 1 multiplexer is used. FSM output *s*_0_ is used as select line and it selects either present or next test pattern bits. For first two clock pulses MSB is not changed. It remains on present test pattern and the third clock pulse new test pattern is applied at MS portion.

BSC generates test vectors which are inserted between consecutive test patterns to reduce vertical transition between consecutive test patterns.

Random bit “*R*” is AND output of present and next test pattern:

(1) $$\begin{array}{c} R=q_{\left(n+1\right)}\;\;\&\;\;q_{\left(n\right)}. \end{array}$$

“*R*” is used to reduce switching transition during test mode. It needs a simple AND gate. In the method proposed in [15] a separate primary input line for random bit insertion is needed. But in this method random bit is generated using present and next test pattern values which avoid the primary input line.

Random bit generated using present and next test pattern also decreases the number of vertical transitions. Random bit generated in this proposed method is either present test pattern bit or next test pattern bit. Therefore the random bit insertion does not increase unnecessary switching transition between two test patterns.

## 4. Implementation of Proposed Method for 8-Bit CUT

**Figure 7:**
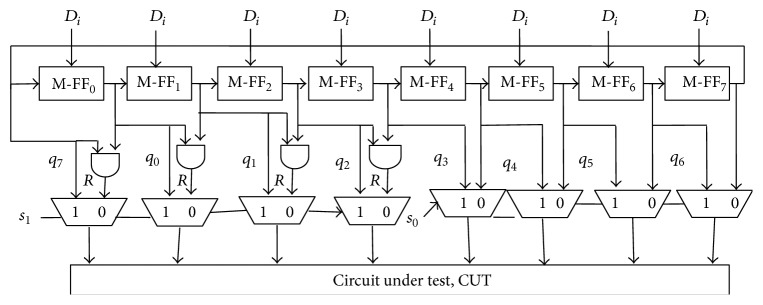
Implementation of proposed method.

An 8-bit CUT requires 8-bit test pattern input during test mode. Therefore proposed TPG is constructed using 8 modified flip flops (see Figure 7). The least four (FF_0_ to FF_3_) flip flop outputs are given to BSC-LSB, and the outputs of FF_4_ to FF_7_ are applied to BSC-MSB (simple MUX) circuit. The bit select circuit outputs are given as test mode inputs to the CUT.

Eight-bit seed is applied from ROM to RC-TPG when the select input line “sel” is “1”. Initially “sel” is asserted and seed is applied from ROM. Then “sel” is changed to logic “0” and ring operation is started which produces test patterns. With particular interval seed should be given to avoid repeated test pattern generation. For each clock a test pattern is generated by the ring counter.

The generated patterns are given to STC to find the total number of switching transitions. If the number of transitions is more test vectors are inserted between test patterns by BS circuit.

For an 8-bit CUT, eight XOR gates, two full adders, and an OR gate are required to construct STC circuit. BSC needs 4 AND gates (for random bit generation) and eight multiplexers (2:1 MUX). FSM has one input line and 3 output lines.

During state B, BSC-LSB replaces the least significant test pattern bits into random bits for test vector generation. The MSB should be frozen for both random bit insertion time and LSB release time. In the third state MSB is changed into next test pattern value.

**Figure 8:**
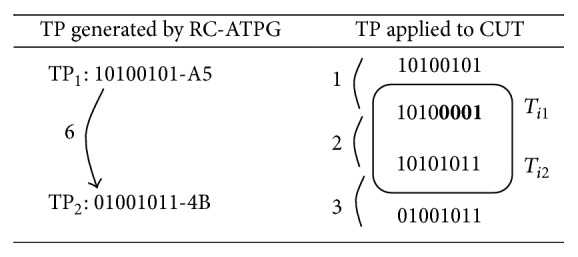
Generated test patterns for 8-bit CUT. TP_1_ and TP_2_: consecutive test patterns; *T*_*i*1_and *T*_*i*2_: test vectors.

Test vectors are formed by MSB of present test pattern and random bits or new test pattern value of LSB. The test vectors are sent to CUT in between two consecutive test patterns which increase the correlation. The patterns with less switching transitions are sent to CUT directly.  Figure 8 shows a small example of inserting test vectors between two consecutive test patterns fed to CUT.

## 5. Experimental Results

The proposed RC-TPG was implemented using Xilinx software (verilog language) in which VCD should be generated after simulation. The obtained results proved that the 50% of dynamic power dissipation is reduced by test vector insertion.

Test patterns sent to CUT have less horizontal and vertical transition than generated test patterns. In between high transition patterns test vectors are inserted (A1 and A5) to reduce number of transitions (see Figure 8). Test vectors are formed using random bits which are shown in bold letters. The new test vector is generated by combining a part from present test pattern and another part from random bit insertion. The random bits reduce transition between consecutive patterns.

Table 3 shows the peak and average power of LFSR and proposed TPG (RC-ATPG) for ISCAS benchmarks. As expected, RC-ATPG reduces the average and peak power. The proposed ATPG reduces up to 38.5% and 50% of the average and peak power, respectively.

In the proposed ATPG external circuit consists of switching transition counter, FSM, and bit selector circuit. The FSM size is fixed.  Table 4 shows the area overhead when RC-TPG is used for test pattern generation. Compared to conventional method the area overhead increases up to 12% and for large circuits it is negligible.

The experimental results (Table 4) clearly show that the proposed method can be implemented for large designs with low area hardware.

## 6. Summary and Conclusion

A novel ATPG using ring counter with reduced dynamic power dissipation is proposed in this paper. The power consumption reduction is achieved by partial insertion of random bits at LS portion and partial freezing of remaining MS bits using external circuit. Adding a simple external circuit (constructed by basic XOR, AND gate, and 2 : 1 multiplexer) the high transition test patterns are identified and the number of vertical transitions is reduced. Ring counter is used as test pattern generator which makes test pattern generation method simple to understand.

Seed is applied in parallel using modified FF which can reduce the time for seed application. Using switching transition counter, less correlative patterns are identified, which avoids unnecessary insertion of test vector in between all consecutive test patterns and reduces testing time. Thus the consecutive test patterns that have few transitions only applied to the CUT. This reduces dynamic power dissipation without affecting the fault coverage. This method proposes a simple ATPG circuit using basic multiplexers and ring counter which generates low transition test patterns. Therefore it can be expanded for large circuits easily.

## Figures and Tables

**Table 1:** Operation performed by modified FF.

Select line	Operation performed
sel = 1	The seed input is accepted from ROM.
sel = 0	Ring operation is performed for test pattern generation.

**Table 2:** Function performed at each FSM state.

FSM state	Select line		Test patterns applied to CUT	
	*s*_1_	*s*_0_	MSB	LSB
A	1	1	Next value	Next value
B	0	0	Present value	Random bit
C	1	0	Present value	Next value

**Table 3:** Power reduction for ISCAS benchmarks.

Circuit	LFSR		RC-ATPG (proposed)	
	*P*_avg_ (*μ*W)	*P*_peak_ (*μ*W)	*P*_avg_ (*μ*W)	*P*_peak_ (*μ*W)
c1908	5.7	26.7	2.8	13.35
c2670	26.4	103.2	12.2	51.6
c3540	12.9	69.6	8.4	34.8
c5315	38.8	219.9	22.6	109.95
s13207	745	4735	602	2367.5
s15850	783	5904	594	2952
s38417	1770	15394	1584	7697
s38584	2466	19880	2102	9940

**Table 4:** Comparison with area overhead.

Circuit	Area overhead (%)		
	LFSR	[15]	RC-ATPG (proposed)
c1908	9.2	11.0	10.2
c3540	10.7	12.3	11.5
s38417	0.7	0.8	0.73
s38584	0.8	0.9	0.83
